# Serum Uric Acid and Microalbuminuria: Predictors of Renal Dysfunction in Type 2 Diabetes Patients in South-Western Uganda

**DOI:** 10.7759/cureus.69843

**Published:** 2024-09-21

**Authors:** Simon Peter Rugera, Jazira Tumusiime, Hope Mudondo, Georgina Naruhura, Ritah Kiconco, Charles Nkubi Bagenda

**Affiliations:** 1 Department of Medical Laboratory Science, Mbarara University of Science and Technology, Mbarara, UGA; 2 Department of Biochemistry, Soroti University, Soroti, UGA

**Keywords:** biomarker, microalbuminuria, renal dysfunction, type 2 diabetes, uric acid

## Abstract

Background

Type 2 diabetes (T2D) is a chronic metabolic disorder characterized by insulin resistance and high blood glucose levels, which has become a global pandemic in recent decades and is associated with several health complications, including renal dysfunction. Serum uric acid levels are associated with kidney damage and have been linked to various health conditions. Urine microalbumin is a sensitive marker of kidney damage and is commonly used to monitor renal dysfunction in diabetes. The study aimed to compare the predictive value of serum uric acid and urine microalbumin in detecting kidney damage among T2D patients.

Method

This secondary data analysis used a cross-sectional dataset of 140 diabetic patients from Mbarara Regional Referral Hospital (MRRH) in Mbarara, Uganda. The main outcome was renal dysfunction, defined as estimated glomerular filtration rate (eGFR) <60 mL/min/1.73m². Key variables included serum uric acid, urinary microalbumin, and various demographic and clinical factors. Data were analyzed using logistic regression and receiver operating characteristic (ROC) curve analysis to evaluate predictive performance. Ethics approval was obtained from the Mbarara University Research Ethics Committee.

Results

This study involved 140 participants with a median age of 53 years (interquartile range (IQR) 44-60.5), predominantly females (95, 67.9%), primarily educated (76, 54.3%), and mostly married (104, 74.3%). Participants with renal dysfunction were older (median age 61 years, IQR 52-69) compared to those without (median age 49, IQR 40-56), with significant differences in urinary microalbumin and serum uric acid levels (p <0.05). Renal dysfunction prevalence was 33.6% (95% CI: 26.2-41.9), higher in participants with diabetes duration ≥5 years, microalbuminuria, certain marital statuses, and higher diastolic blood pressure. Microalbuminuria (adjusted odds ratio (aOR) 4.71, 95% CI: 1.27-17.50, P = 0.021) and serum uric acid (aOR 1.01, 95% CI: 1.0002-1.0153, P = 0.045) were significantly associated with renal dysfunction. Other associated factors included age, female gender, and diastolic hypertension. Both biomarkers had significant predictive power for renal dysfunction (area under the curve (AUC) 0.62 and 0.65, respectively).

Conclusion

This study confirms the high prevalence of renal dysfunction among T2D patients, with a finding of 33.6%. The significant association between microalbuminuria and renal dysfunction, as well as the predictive capacity of serum uric acid and urinary microalbumin, highlight the importance of these biomarkers in identifying individuals at risk of kidney complications.

## Introduction

Type 2 diabetes (T2D) is a chronic metabolic disorder characterized by insulin resistance and high blood glucose levels [1]. It is the most common and clinically important metabolic disorder, which has become a global pandemic in recent decades and a major healthcare burden worldwide [2]. Rapid economic development and urbanization have led to a rising burden of diabetes in many parts of the world [3]. According to the World Health Organization, more than 420 million people around the world were living with diabetes in 2021, a number that is expected to rise to 578 million by 2030 [4]. The overall prevalence of diabetes mellitus (DM) in Uganda has been established at 1.4% [5], with the highest prevalence in urban communities [3]. Increased consumption of unhealthy diets and sedentary lifestyles, resulting in elevated body mass index (BMI) and fasting plasma glucose, have been blamed for these trends [6]. Type 2 diabetes has been associated with several health complications, including renal dysfunction [7].

Renal dysfunction is a significant complication of T2D that contributes to increased morbidity and mortality among patients [8]. The consequences of renal dysfunction range from chronic kidney disease and acute kidney injury to end-stage renal disease [9]. The early identification and management of renal dysfunction in T2D patients is crucial for improving patient outcomes and reducing the burden on healthcare systems [10]. Patients with end-stage renal disease have reduced quality of life, high levels of morbidity, and a mortality of about 10.3% [11]. Given the high morbidity and mortality rates among these patients, it is essential to identify effective methods for predicting and managing renal dysfunction in T2D. Decreased glomerular filtration rate (GFR), elevated serum creatinine and uric acid levels, and electrolyte imbalances are key features in the laboratory diagnosis of renal dysfunction here in Uganda [12].

Serum uric acid is the end product of purine metabolism in humans and has been implicated in various health conditions, including hypertension, chronic kidney disease, T2DM, and cardiovascular disease [13]. The association between hyperuricemia (uric acid levels >7 mg/dL) and renal dysfunction has been observed in previous studies, suggesting that uric acid may play a role in the pathophysiology of kidney disease [14]. However, the use of serum uric acid as a predictive marker for renal dysfunction in T2D patients remains controversial and inconclusive.

Urine microalbumin (≥ 30 mg/day) is produced from the small leakage of albumin from the glomeruli of the kidney [15]. Elevated levels of urine microalbumin have been associated with early kidney damage and can serve as an indicator of renal dysfunction in T2D [16]. Urine microalbumin is a sensitive marker of kidney damage and is commonly used to monitor renal dysfunction in diabetes. Hence, if no interventions are made, the urinary albumin excretions increase, and patients eventually end up with end-stage renal disease [17]. A study by Warjukar et al. identified the usefulness of microalbuminuria as an early detector of diabetic nephropathy [18]. In the same study, uric acid levels were seen to provide an early screen for diabetic nephropathy in the same patients. However, further research is needed to evaluate its predictive value in detecting renal dysfunction among T2D patients. In our study, we aimed to compare the predictive value of serum uric acid and urine microalbumin.

## Materials and methods

Study design, setting and population, and eligibility criteria

This study was a secondary data analysis of a dataset from a cross-sectional study conducted among diabetic patients at Mbarara Regional Referral Hospital (MRRH) [12]. The title of the cross-sectional study whose dataset has been used for secondary analysis is “Microalbuminuria and Traditional Serum Biomarkers of Nephropathy among Diabetic Patients at Mbarara Regional Referral Hospital in South Western Uganda” [12]. The hospital is located in Mbarara city, South Western Uganda, and is approximately 260 km from Kampala, the capital city. All patients enrolled in the cross-sectional study were already diagnosed with either Type 1 or Type 2 diabetes mellitus, receiving treatment from the diabetic clinic of MRRH, and had provided written informed consent. Pregnant women and patients with other medical kidney diseases were excluded from participating. One hundred and forty diabetic patients who satisfied the inclusion criteria were enrolled in the cross-sectional study.

Sample size and sampling technique

A sample size of 140 study participants was established in the primary cross-sectional study, whose major variable was microalbuminuria. Sampling of study participants in the primary study was done consecutively until the required sample size was obtained.

Variables and equipment

The major outcome variable in the primary cross-sectional study was microalbuminuria, which was defined as a urine albumin concentration of 2-20 mg/dL [12]. In this secondary data analysis study, the main outcome variable is renal dysfunction, which is defined as an estimated glomerular filtration rate (eGFR) of <60 ml/min/1.73 m^2^ [19]. As per the Kidney Foundation’s Kidney Disease Outcome Quality Initiative (K/DOQI), eGFR<60 ml for three months implies chronic kidney disease [20]. The eGFR was calculated as reported by Levey et al. [21]. Serum uric acid levels and urinary microalbumin are the main independent variables whose association with renal dysfunction and the predictive performance in the discrimination of participants with and without renal dysfunction were being investigated. The other independent variables in the dataset whose relationship with renal dysfunction was being investigated included age, gender, marital status, education level, smoking status, alcohol consumption status, blood pressure, hypertension, family history of diabetes mellitus, duration with diabetes mellitus, serum levels of sodium, potassium, and chloride, and glucose.

In the primary study [12], pretested questionnaires were used to obtain participants’ age, sex, duration with DM, and lifestyle risk factors, e.g., smoking and alcohol consumption. Blood pressure was measured on the day of enrolment in the study using a mercury sphygmomanometer with small (<21 cm) and normal (22-32 cm) cuff sizes on the left arm at the level of the heart while the patient was seated. Hypertension was defined as systolic blood pressure ≥140 and/or ≥90 mmHg or use of hypertensive medication. The participant’s weight was measured using a weighing scale machine (Seca, Seca GmbH & Co. KG., Hamburg, Germany), making sure that the patient had no heavy clothing or shoes. Height was measured to the nearest 0.1 cm against a vertical wall. The participant’s weight in kilograms and the square of their height in meters were used to calculate their BMI. The details on the urine and blood collection, storage, and clinical chemistry analyzers used in the quantitative determination of urinary microalbumin and serum creatinine, electrolytes, and glucose are indicated in the primary publication [12].

Statistical analysis

Data from Microsoft Excel (Microsoft Corp., Redmond, WA) was imported into STATA software, version 17 (StataCorp LLC, College Station, TX) for cleaning and analysis. All the continuous variables (age, systolic and diastolic blood pressure, BMI, and serum parameters of sodium, potassium, chloride, creatinine, and uric acid) were tested for normality using the Shapiro-Wilk normality test. Serum sodium and uric acid levels that were normally distributed across the study participants were summarized using the mean together with the standard deviation, while the other continuous variables that were not normally distributed were summarized using the median with the interquartile range (IQR). The mean levels of uric acid and sodium were compared between the study participants with renal dysfunction and those without renal dysfunction using a student T-test to test for any statistical difference. For the variables with a skewed distribution, medians were compared using the Wilcoxon rank sum test. A p-value <0.05 was considered statistically significant. As for this study, eGFR was categorized into renal dysfunction present (eGFR <60 mL/min/1.73 m^2^) and kidney dysfunction absent [19]. The continuous variables body mass index, glucose, urinary microalbumin, sodium, potassium, and chloride were also further categorized using the cut-off points and reference ranges as clearly described in the primary publication [12]. All categorical variables were summarized using frequencies and percentages.

The prevalence of renal dysfunction together with its 95% confidence interval (CI) was obtained by dividing the number of participants with renal dysfunction by the total number of participants that were recruited in the study (sample size) and was expressed as a percentage. The distribution of renal dysfunction prevalence in the different levels of each of the categorical variables was compared using a chi-square test or the Fisher’s exact test to test if there is a significant difference in the prevalence distribution in the different levels. A p-value <0.05 was considered statistically significant.

To assess the relationship between the main independent variables (microalbuminuria and serum uric acid levels) and the main binary (1,0) outcome variable (renal dysfunction), we used logistic regression. Participants who had eGFR <60mL/min/1.73m^2^ were considered to have renal dysfunction (1), whereas those with eGFR >60mL/min/1.73m^2^ were considered not to have renal dysfunction (0). Each of the independent variables at the bivariate level was compared with renal dysfunction. The associations were determined using crude odds ratios (cOR) together with their 95% CI, and the statistically significant odds ratios (ORs) were indicated by a p-value less than 0.2 at the bivariate level. The variables that were clinically and/or statistically significant at this level were also included in the multivariable model to adjust for confounding effects. The full multivariate model was adjusted via the removal of some of the non-significant variables to improve the model's precision without significant loss of validity in predicting renal dysfunction. The Hosmer-Lemeshow test was used to test the suitability of the final model in predicting renal dysfunction. In the final multivariable model, associations were determined using adjusted odds ratios (aOR) together with their 95% CI. Associations were considered significant at a p-value ≤0.05.

To assess the predictive performance of serum uric acid and urinary microalbumin for renal dysfunction, we used receiver operating characteristic (ROC) curve analysis. Predictive power was evaluated using the area under the curve (AUC) and its 95% CI; a value near one indicates better performance and a 95% CI that included 0.5 indicates a non-significant ability in discriminating between those with renal dysfunction and those without renal dysfunction. We used Youden's index to estimate the optimal cutoff point of the two biomarkers for dysfunction prediction at maximum sensitivity and specificity.

Ethical considerations

Permission was granted by the principal investigator, Ritah Kiconco, of the primary study whose dataset was used for this study. Ethical clearance was sought from the Research Ethics Committee (REC) of Mbarara University of Science and Technology to perform this secondary data analysis study (approval number 36/05-17/MUST-2023-1039).

## Results

Sociodemographic characteristics of the study participants

A total of 140 study participants were recruited in the primary study. The median age of the study participants was 53 years with an IQR of 44 years to 60.5 years, with the majority of the participants being in the age group 40 to 45 years. The median age was significantly higher in the study participants with renal dysfunction (61 years, IQR: 52-69) in comparison to that observed in those participants without renal dysfunction (49 years, IQR: 40-56, p-value <0.001). The majority of the study participants were female (95, 67.9%), attained a primary level of education (76, 54.3%), and were married (104, 74.3%) (Table 1). The two groups of the outcome variable were not significantly different from each other in the distribution of participants’ social demographic characteristics with the exception of age and marital status. Significant differences in the levels of the primary biomarkers (urinary microalbumin and serum uric acid) were observed between the study participants who had renal dysfunction and those who did not (p-value <.05), with higher levels noted in the participants with renal dysfunction as indicated in Table 1. The levels of other serum biomarkers were not statistically different in those with and without renal dysfunction (Table 1).

**Table 1:** Characteristics of study participants stratified by renal dysfunction Chi-square test, Fisher’s exact test, Student’s t-test, and Wilcoxon rank-sum test were used to obtain the p-values

Variable	Total n=140(100%)	Renal dysfunction		p-value
		Present 47(33.6%)	Absent 94(66.4%)	
Gender				0.050
Male	45(32.1)	10(22)	35(78)	
Female	95(67.9)	37(39)	58(61)	
Age (in years)				<0.001
18-34	13(9.3)	0(0)	13(100)	
35-44	25(17.9)	1(4)	24(96)	
45-54	41(29.3)	14(34)	27(66)	
55-64	36(25.7)	13(36)	23(64)	
> /=65	25(17.9)	19(76)	6(24)	
Age: median (interquartile range (IQR))	53(44-60.5)	61(52-69)	49(40-56)	<0.001
Marital status				0.005
Married	104(74.3)	28(26.9)	76(73.1)	
Single	36(25.7)	19(52.8)	17(47.2)	
Education level				0.20
College	17(12.1)	3(18)	14(82)	
Never went to school	18(12.9)	8(44)	10(56)	
Primary	76(54.3)	29(38)	47(62)	
Secondary	29(20.7)	7(24)	22(76)	
Systolic blood pressure (BP)				0.85
Absent	61(43.6)	21(34)	40(66)	
Present	79(56.4)	26(33)	53(67)	
Diastolic BP				0.047
Absent	119(85)	44(37.0)	75(63.0)	
Present	21(15)	3(14.3)	18(85.7)	
Hypertension				0.081
No	59(42.1)	15(25)	44(75)	
Yes	81(57.9)	32(40)	49(60)	
Combined BP categories (in mmHg)				0.42
Normal	56(40)	21(38)	35(63)	
Hypertensive	84(60)	26(31)	58(69)	
Systolic BP	140(132-147)	140(132-147)	140(132-147)	0.78
Diastolic BP	78(70-84.5)	76(70-85)	78(72-84)	0.20
Smoking				0.26
No	137(97.9)	45(34.4)	92(67.2)	
Yes	3(2.1)	2(66.7)	1(33.3)	
Alcohol				0.72
No	131(93.6)	45(34.4)	86(65.6)	
Yes	9(6.4)	2(22.2)	7(77.8)	
BMI categories				1.00
<25kg/m^2^	84(60)	28(33)	56(67)	
25-29.9 kg/m^2^	49(35)	17(35)	32(65)	
>=30kg/m2	7(5)	2(29)	5(71)	
BMI (kg/m^2^)	24.26717(22.1-26.4)	24.048(22.024-26.298)	24.314(22.024-26.298)	0.43
Diabetes mellitus (DM) duration				0.036
<=5 years	74(52.9)	19(26)	55(74)	
>5years	66(47.1)	28(42)	38(58)	
Family history of DM				0.49
No	109(77.9)	35(32.1)	74(67.9)	
Yes	31(22.1)	12(38.7)	19(61.3)	
Microalbuminuria				0.014
Absent	103(76.9)	26(25.2)	77(74.8)	
Present	31(23.1)	15(48.4)	16(51.6)	
Microalbuminuria	0.83(0.44-1.98)	1.29(0.67-3.06)	0.68(0.41-1.45)	0.006
Electrolytes imbalance				0.98
Absent	30(21.4)	10(33.3)	20(66.7)	
Present	110(78.6)	37(33.6)	73(66.4)	
Dysglycaemia				0.68
In control	51(36.4)	16(31)	35(69)	
Out of control	89(63.6)	31(35)	58(65)	
Glucose (mmol/L)	7.845(5.4-11.685)	7.65(6-12.29)	8.6(4.92-11.32)	0.53
Uric acid (mg/dL)	3.923(3.346-5.139)	4.660(3.388-5.624)	3.770(3.327-4.681)	0.038
Creatinine (umol/L)	107.55(95.8-118.15)	117 (111.9-132.8)	99.1 (91.6-110.2)	<0.001
Sodium (mEq/L)	153.830(8.524)	154.61362 (7.91)	153.4343 (8.83)	0.44
Potassium (mEq/L)	4.78(4.25-5.2)	4.6 (4.4-5.2)	4.8 (4.2-5.2)	0.66
Chloride (mmol/L)	97.37(90.82-105.26)	97.37(91.84-105.26)	97.37(89.8-105.26)	0.74
Sodium/potassium ratio	32.43(29.30-36.64)	33.13(29.54-6.46)	32.17(29.19-36.82)	0.42
Dysnatremia				0.94
Normal levels	84(60)	28 (33%)	56 (67%)	
Hypernatremia	56(40)	19 (34%)	37 (66%)	
Dyskalemia				0.62
Normal levels	138(98.6)	46 (33.3%)	92 (66.7%)	
Hyperkalemia	2(1.4)	1 (50.0%)	1 (50.0%)	
Dyschloraemia				0.90
Normal levels	53(37.9)	19 (36%)	34 (64%)	
Hyperchloremia	22(15.7)	7 (32%)	15 (68%)	
Hypochloremia	65(46.4)	21 (32%)	44 (68%)	

Prevalence of renal dysfunction

**Figure 1:**
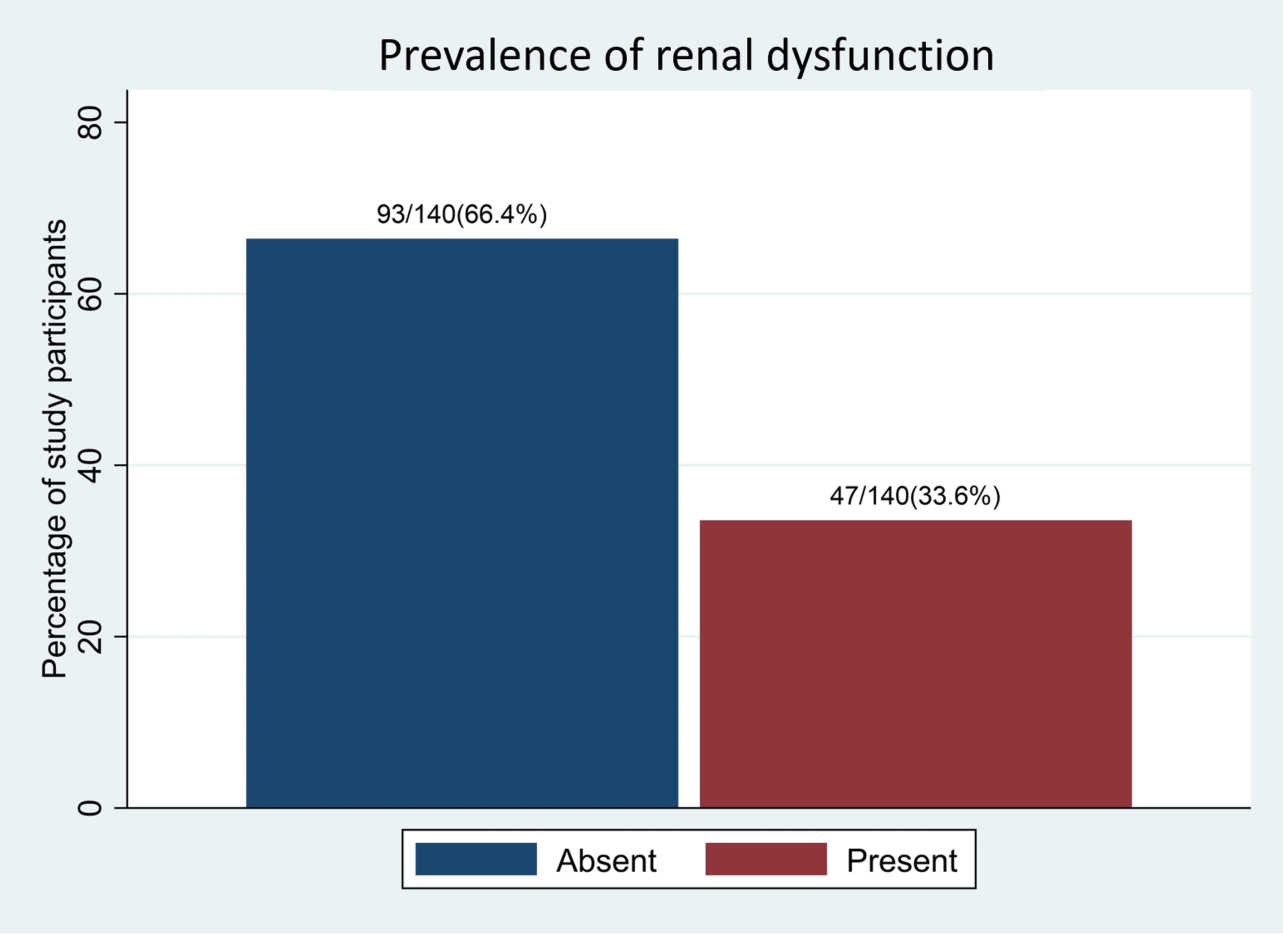
Prevalence of renal dysfunction among type 2 diabetes mellitus patients

Out of the 140 study participants, 67 had eGFR <60 ml/min/1.73 m^2^, giving an overall renal dysfunction prevalence of 47 (33.6%) (95%CI: 26.2 - 41.9), as indicated in Figure 1. We observed a significantly higher prevalence of renal dysfunction among participants with DM duration of ≥5 years (28, 42%), compared to the prevalence of 19 (26%) in participants with DM duration of <5 years (p-value 0.036). A significantly higher prevalence (15, 48.4%) was also observed among study participants with microalbuminuria in comparison to the group that did not have microalbuminuria (26, 25.2%, p-value 0.014). Other categorical variables whose levels showed significant differences in the distribution of renal dysfunction prevalence include marital status and diastolic blood pressure, as indicated in Table 1.

Association between serum uric acid and urinary microalbumin with renal dysfunction

After controlling for confounding effects of other variables present in the dataset, the odds of renal dysfunction were 4.71 times higher in study participants who had microalbuminuria than the odds in those without (aOR 4.71 (95%CI: 1.27-17.50, p-value 0.021)). High serum uric acid levels were also significantly associated with higher odds of renal dysfunction (aOR: 1.01 (95%CI: 1.0002 1.0153, p-value 0.045)), as indicated in Table 2. Other variables that showed a significant association with renal dysfunction were age (aOR: 1.17 (95% CI: 1.09 1.25, p-value <0.001)), female gender (aOR: 3.89 (95% CI: 1.17 13.04, p-value 0.028)), and having diastolic hypertension (aOR: 0.10 (95% CI: 0.01 0.74, p-value 0.024)) (Table 2).

**Table 2:** Association between serum uric acid and urinary microalbumin with renal dysfunction Logistic regression analysis was used to obtain the p-values. An odds ratio of 1.00 indicates the odds of the outcome variable (renal dysfunction) in a reference group when it is compared against itself. cOR: crude odds ratio; aOR: adjusted odds ratio; DM: diabetes mellitus

Variable	Bivariate analysis		Multivariate analysis	
	cOR(95%CI)	p-value	aOR(95%CI)	p-values
Age	1.12(1.07-1.16)	<0.001	1.17(1.09-1.25)	<0.001
Gender				
Male	1.00	-	1.00	-
Female	2.23(0.99-5.04)	0.053	3.89(1.17-13.04)	0.028
Microalbuminuria				
Absent	1.00	-	1.00	-
Present	2.78(1.21-6.39)	0.016	4.71(1.27-17.50)	0.021
Serum uric acid	1.01(1.001-1.011)	0.023	1.01(1.0002-1.0153)	0.045
Marital status				
Married	1.00	-	-	-
Single	3.03(1.38-6.65)	0.006	-	-
Education level				
Never went to school	1.00	-	-	-
Primary	0.77(0.27-2.18)	0.624	-	-
College	0.27(0.06-1.27)	0.097	-	-
Secondary	0.40(0.11-1.40)	0.152	-	-
Systolic hypertension				
Absent	1.00	-	1.00	-
Present	0.93(0.46-1.90)	0.851	1.31(0.39-4.42)	0.668
Diastolic hypertension				
Absent	1.00	-	1.00	-
Present	0.28(0.08-1.02)	0.054	0.10(0.01- 0.74)	0.024
Hypertension				
No	1.00	-	1.00	-
Yes	1.92(0.92-3.99)	0.083	0.49(0.15-1.62)	0.241
Smoking				
No	1.00	-	1.00	-
Yes	4.09(0.36-46.30)	0.255	0.51(0.01-19.82)	0.716
Alcohol				
No	1.00	-	1.00	-
Yes	0.55(0.11-2.74)	0.462	0.56(0.07-4.42)	0.579
BMI				
<25kg/m2	1.00	-	1.00	-
25-29.9 kg/m2	1.06(0.51-2.23)	0.873	1.15(0.36-3.65)	0.816
≥30kg/m2	0.80(0.15-4.39)	0.797	1.51(0.14-16.59)	0.737
DM duration				
≤5 years	1.00	-	1.00	-
>5years	2.13(1.04-4.36)	0.038	0.92(0.32-2.70)	0.885
Family history of DM				
No	1.00	-	1.00	-
Yes	1.34(0.58-3.05)	0.493	2.45(0.69-8.75)	0.167
Sodium/potassium ratio	1.01(0.96-1.08)	0.628	1.06(0.95-1.17)	0.305
Electrolytes imbalance				
Absent	1.00	-	-	-
Present	1.01(0.43-2.39)	0.975	-	-
Dysglycaemia				
In control	1.00	-	1.00	-
Out of control	1.17(0.56-2.44)	0.677	0.87(0.27-2.81)	0.813
Dysnatremia				
Normal levels	1.00	-	1.00	-
Hypernatremia	1.03(0.50-2.10)	0.942	2.43(0.78-7.54)	0.124
Dyskalemia				
Normal levels	1.00	-	1.00	-
Hyperkalemia	2.00(0.12-32.70)	0.627	1.46(0.04-57.58)	0.840
Dyschloremia				
Normal levels	1.00	-	1.00	-
Hyperchloremia	0.84(0.29-2.41)	0.739	0.83(0.15-4.58)	0.827
Hypochloremia	0.85(0.40-1.84)	0.686	1.28(0.40-4.10)	0.675

**Figure 2:**
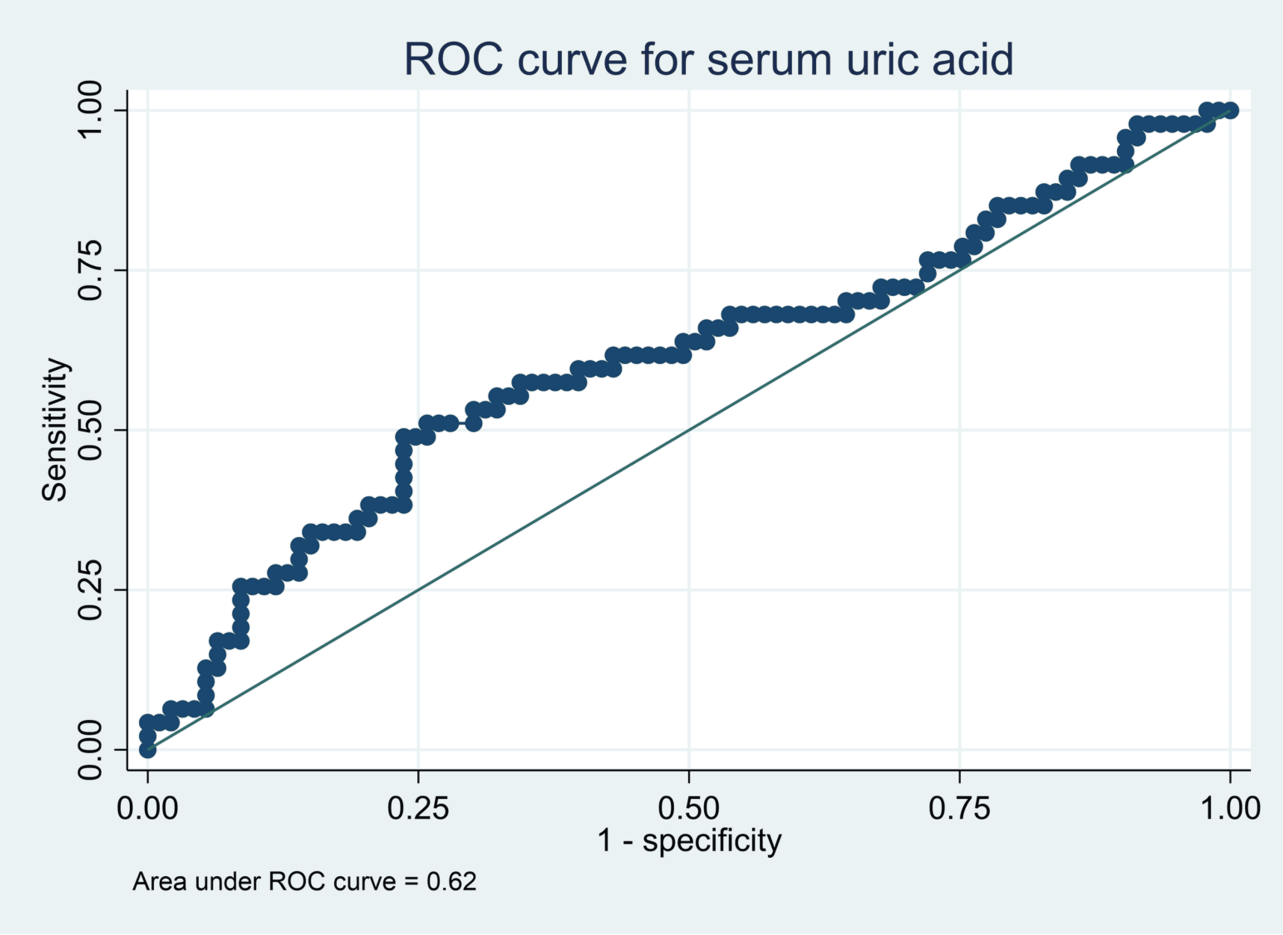
Predictive performance for serum uric acid ROC: receiver operating characteristic

**Figure 3:**
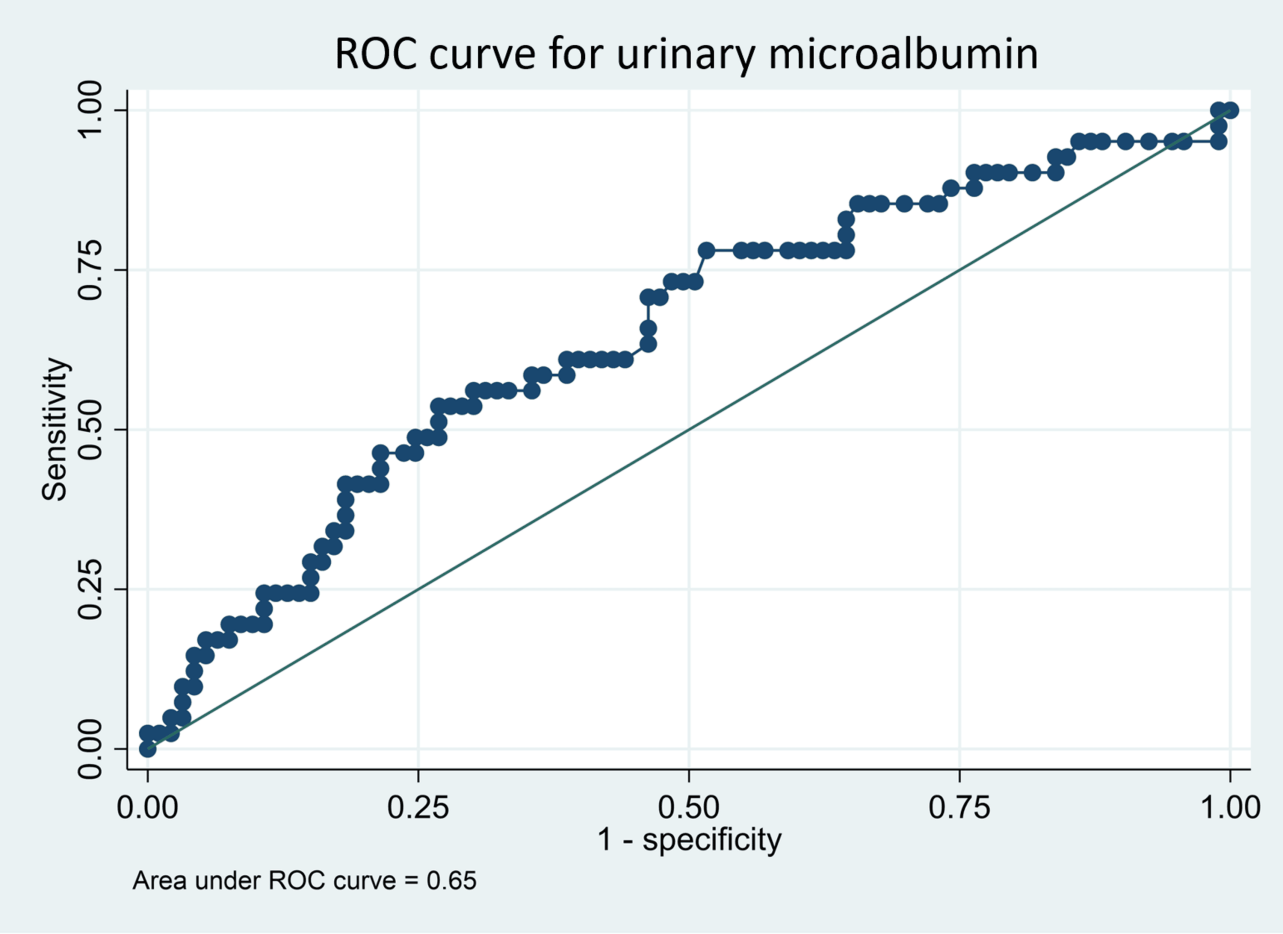
Predictive performance for urinary microalbumin ROC: receiver operating characteristic

**Figure 4:**
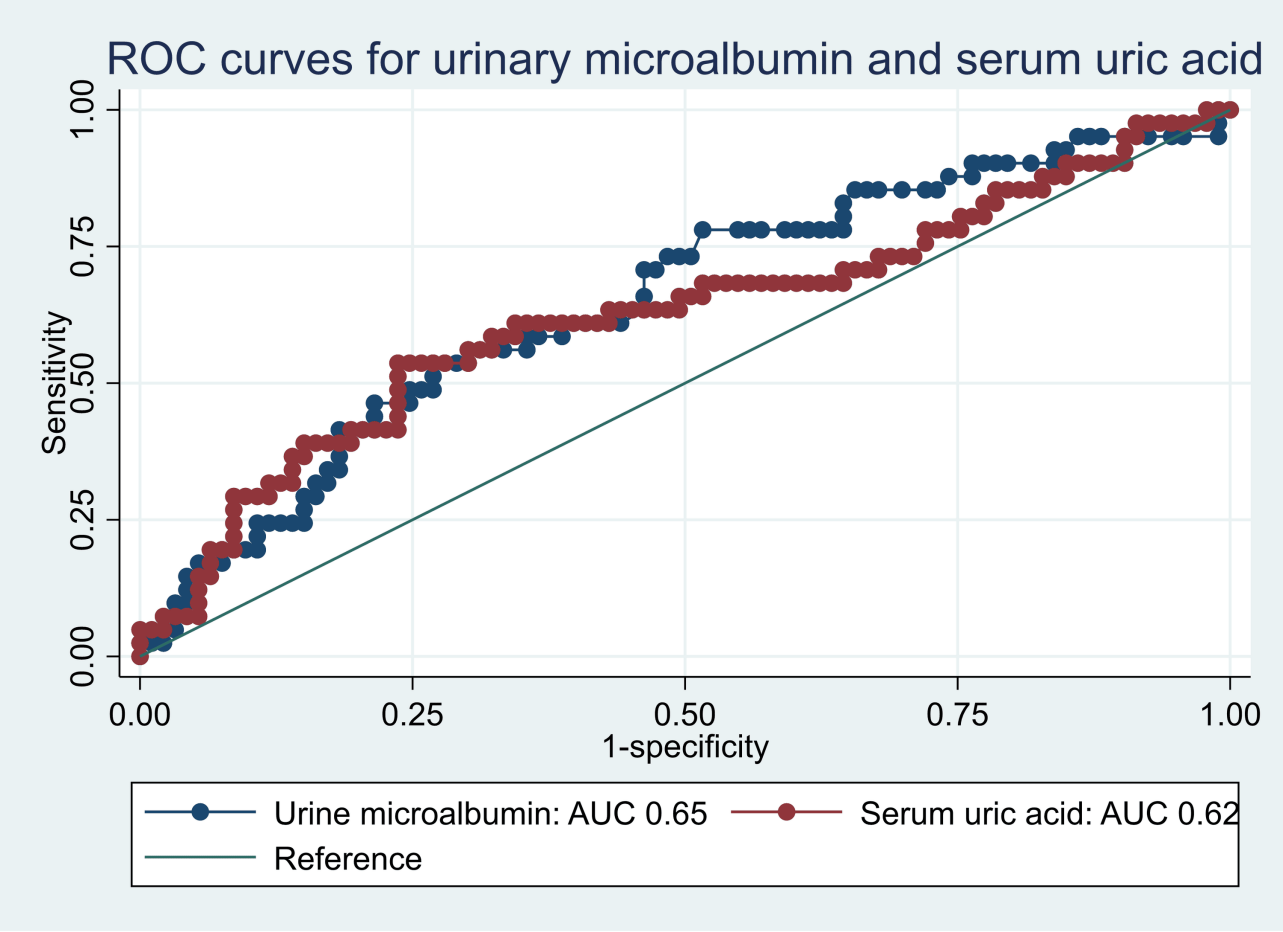
Comparison of the predictive performances for serum uric acid and urinary microalbumin ROC: receiver operating characteristic; AUC: area under the curve

Both serum uric acid (AUC: 0.62, 95% CI: 0.51-0.73) and urinary microalbumin (AUC: 0.65, 95% CI: 0.55-0.75) had significant predictive power to discriminate participants with renal dysfunction from those participants without at their respective optimal cut-off points at maximum sensitivity and specificity as indicated in Table 3 and Figures 2-4. The two biomarkers did not significantly differ from each other in terms of their predictive ability for renal dysfunction (AUCs 0.62 vs. 0.65, p-value 0.7308).

**Table 3:** Predictive performance of urinary microalbumin and serum uric acid for renal dysfunction AUC: area under the curve

Variable	AUC	95% CI	Optimal cutoff	Sensitivity (%)	Specificity (%)	Youden's J index
Serum uric acid	0.62	0.51-0.73	≥285.65	49	76	0.253
Urinary micro albumin	0.65	0.55-0.75	≥ 11.80	54	73	0.090

## Discussion

The prevalence of renal dysfunction among T2D patients was 33.6%. This finding is consistent with previous research, which reported a prevalence of 29% renal impairment among T2D patients [22]. Additionally, studies have shown that approximately 20% of adult T2D patients have an eGFR of less than 60 mL/min/1.73 m^2^ [23]. The prevalence of renal dysfunction in the US diabetic patient population has been reported to range from 30% to 50%, which aligns with our findings [24]. Furthermore, Shahwan et al. observed a higher prevalence of 42% renal dysfunction among a diabetic population, which is higher than our study population [25]. These findings collectively emphasize the high prevalence of renal dysfunction among T2D populations, underscoring the need for effective strategies to address renal complications in diabetic patients [26].

Our study revealed a statistically significant disparity in the prevalence of microalbuminuria between individuals without (25.2%) and those with (48.4%) microalbuminuria. Notably, those with microalbuminuria exhibited a significantly higher risk of developing renal dysfunction, with an aOR of 4.71 (95% CI: 1.27, 17.50, P = 0.021). This finding is consistent with previous research, which has consistently demonstrated an association between microalbuminuria and renal dysfunction in diabetic patients [27, 28].

The analysis also uncovered significant correlations between elevated serum uric acid levels, urinary microalbumin, and renal dysfunction. These biomarkers emerged as potential predictors for identifying renal dysfunction in T2D patients. The observed higher levels of serum uric acid and urinary microalbumin in participants with renal dysfunction support their relevance in predicting kidney complications among diabetic individuals. These findings align with previous research suggesting the utility of these biomarkers in assessing renal health in T2DM [29]. Specifically, Han et al. reported an odds ratio of 1.005 for the association between uric acid and renal dysfunction is consistent with our observed associations [29].

This investigation demonstrated notable predictive capacity to differentiate subjects with renal dysfunction from those without it for serum uric acid and urinary microalbumin. Consistent with previous research by Han et al. and Afifa et al., our findings underscore the utility of these biomarkers in identifying individuals with renal dysfunction [29, 30]. Notably, however, the predictive power of serum uric acid and urinary microalbumin did not exhibit statistically significant differences in our study.

However, this study is a secondary data analysis of a primary study's data, so we had limited control over the data collection process. We also used data from a cross-sectional study for secondary analysis, and therefore the exposure-effect relationship remains unclear. Future studies incorporating larger sample sizes and longitudinal designs could provide more robust evidence regarding the reliability and specificity of these biomarkers in predicting renal dysfunction among T2DM patients.

## Conclusions

In conclusion, our study confirms the high prevalence of renal dysfunction among T2D patients, with a finding of 33.6% consistent with previous research. The significant association between microalbuminuria and renal dysfunction, as well as the predictive capacity of serum uric acid and urinary microalbumin, underscore the importance of these biomarkers in identifying individuals at risk of kidney complications. These findings align with previous research and emphasize the need for effective strategies to address renal complications in diabetic patients. Future studies with larger sample sizes and longitudinal designs will provide more robust evidence regarding the reliability and specificity of these biomarkers in predicting renal dysfunction among type 2 diabetes mellitus patients.

## References

[1] Hameed I, Masoodi SR, Mir SA, Nabi M, Ghazanfar K, Ganai BA (2015). Type 2 diabetes mellitus: from a metabolic disorder to an inflammatory condition. World J Diabetes.

[2] Reed J, Bain S, Kanamarlapudi V (2021). A review of current trends with type 2 diabetes epidemiology, aetiology, pathogenesis, treatments and future perspectives. Diabetes Metab Syndr Obes.

[3] Onyango EM, Onyango BM (2018). The rise of noncommunicable diseases in Kenya: an examination of the time trends and contribution of the changes in diet and physical inactivity. J Epidemiol Glob Health.

[4] (2021 ). New WHA Resolution to bring much needed boost to diabetes prevention and control efforts. Diabetes Prevention and Control Efforts.

[5] Bahendeka S, Wesonga R, Mutungi G, Muwonge J, Neema S, Guwatudde D (2016). Prevalence and correlates of diabetes mellitus in Uganda: a population-based national survey. Trop Med Int Health.

[6] Lone S, Lone K, Khan S, Pampori RA (2017). Assessment of metabolic syndrome in Kashmiri population with type 2 diabetes employing the standard criteria's given by WHO, NCEPATP III and IDF. J Epidemiol Glob Health.

[7] Evans M, Morgan AR, Patel D (2021). Risk prediction of the diabetes missing million: identifying individuals at high risk of diabetes and related complications. Diabetes Ther.

[8] Penno G, Solini A, Bonora E (2018). Defining the contribution of chronic kidney disease to all-cause mortality in patients with type 2 diabetes: the Renal Insufficiency And Cardiovascular Events (RIACE) Italian Multicenter Study. Acta Diabetol.

[9] Campos P, Ortiz A, Soto K (2016). HIV and kidney diseases: 35 years of history and consequences. Clin Kidney J.

[10] Shlipak MG, Tummalapalli SL, Boulware LE (2021). The case for early identification and intervention of chronic kidney disease: conclusions from a Kidney Disease: Improving Global Outcomes (KDIGO) controversies conference. Kidney Int.

[11] Ma SJ, Wang WJ, Tang M, Chen H, Ding F (2021). Mental health status and quality of life in patients with end-stage renal disease undergoing maintenance hemodialysis. Ann Palliat Med.

[12] Kiconco R, Rugera SP, Kiwanuka GN (2019). Microalbuminuria and traditional serum biomarkers of nephropathy among diabetic patients at Mbarara Regional Referral Hospital in south western Uganda. J Diabetes Res.

[13] Furuhashi M (2020). New insights into purine metabolism in metabolic diseases: role of xanthine oxidoreductase activity. Am J Physiol Endocrinol Metab.

[14] Srivastava A, Kaze AD, McMullan CJ, Isakova T, Waikar SS (2018). Uric acid and the risks of kidney failure and death in individuals with CKD. Am J Kidney Dis.

[15] Comper WD, Hilliard LM, Nikolic-Paterson DJ, Russo LM (2008). Disease-dependent mechanisms of albuminuria. Am J Physiol Renal Physiol.

[16] Thakur V, Chattopadhyay M (2018). Early urinary markers for diabetic and other kidney diseases. Curr Drug Targets.

[17] AlFehaid AA (2017). Prevalence of microalbuminuria and its correlates among diabetic patients attending diabetic clinic at National Guard Hospital in Alhasa. J Family Community Med.

[18] Warjukar P, Jain P, Kute P, Anjankar A, Ghangale SS (2020). Study of microalbuminuria and uric acid in type 2 diabetes mellitus. Int J Cur Res Rev.

[19] Nyende L, Kalyesubula R, Sekasanvu E, Byakika-Kibwika P (2020). Prevalence of renal dysfunction among HIV infected patients receiving Tenofovir at Mulago: a cross-sectional study. BMC Nephrol.

[20] Wouters OJ, O'Donoghue DJ, Ritchie J, Kanavos PG, Narva AS (2015). Early chronic kidney disease: diagnosis, management and models of care. Nat Rev Nephrol.

[21] Levey AS, Becker C, Inker LA (2015). Glomerular filtration rate and albuminuria for detection and staging of acute and chronic kidney disease in adults: a systematic review. JAMA.

[22] Retnakaran R, Cull CA, Thorne KI, Adler AI, Holman RR (2006). Risk factors for renal dysfunction in type 2 diabetes: U.K. prospective Diabetes Study 74. Diabetes.

[23] Thomas MC, Cooper ME, Zimmet P (2016). Changing epidemiology of type 2 diabetes mellitus and associated chronic kidney disease. Nat Rev Nephrol.

[24] Gheith O, Farouk N, Nampoory N, Halim MA, Al-Otaibi T (2015). Diabetic kidney disease: world wide difference of prevalence and risk factors. J Nephropharmacol.

[25] Jamal Shahwan M, Hassan NA, Shaheen RA (2019). Assessment of kidney function and associated risk factors among type 2 diabetic patients. Diabetes Metab Syndr.

[26] Elhefnawy KA, Elsayed AM (2019). Prevalence of diabetic kidney disease in patients with type 2 diabetes mellitus. Egypt J Intern Med.

[27] Markus MR, Ittermann T, Baumeister SE (2018). Prediabetes is associated with microalbuminuria, reduced kidney function and chronic kidney disease in the general population: The KORA (Cooperative Health Research in the Augsburg Region) F4-Study. Nutr Metab Cardiovasc Dis.

[28] Ali A, Taj A, Amin MJ, Iqbal F, Iqbal Z (2014). Correlation between microalbuminuria and hypertension in type 2 diabetic patients. Pak J Med Sci.

[29] Han R, Duan L, Zhang Y, Jiang X (2023). Serum uric acid is a better indicator of kidney impairment than serum uric acid-to-creatinine ratio and serum uric acid-to-high-density lipoprotein ratio: a cross-sectional study of type 2 diabetes mellitus patients. Diabetes Metab Syndr Obes.

[30] Afifa K, Belguith Asma S, Nabil H (2016). Screening for nephropathy in diabetes mellitus: is micral-test valid among all diabetics?. Int J Chronic Dis.

